# Role of Aβ in Alzheimer’s-related synaptic dysfunction

**DOI:** 10.3389/fcell.2022.964075

**Published:** 2022-08-26

**Authors:** Huiqin Zhang, Xuefan Jiang, Lina Ma, Wei Wei, Zehui Li, Surui Chang, Jiayu Wen, Jiahui Sun, Hao Li

**Affiliations:** ^1^ Institute of Geriatrics, Xiyuan Hospital, China Academy of Chinese Medical Sciences, Beijing, China; ^2^ Beijing University of Chinese Medicine, Beijing, China; ^3^ Wangjing Hospital, China Academy of Chinese Medical Sciences, Beijing, China

**Keywords:** synaptic dysfunction, Alzheimer’s disease, β-amyloid, glutamate receptors, synaptic plasticity, synapse toxicity

## Abstract

Synaptic dysfunction is closely related to Alzheimer’s disease (AD) which is also recognized as synaptic disorder. β-amyloid (Aβ) is one of the main pathogenic factors in AD, which disrupts synaptic plasticity and mediates the synaptic toxicity through different mechanisms. Aβ disrupts glutamate receptors, such as NMDA and AMPA receptors, which mediates calcium dyshomeostasis and damages synapse plasticity characterized by long-term potentiation (LTP) suppression and long-term depression (LTD) enhancement. As Aβ stimulates and Ca^2+^ influx, microglial cells and astrocyte can be activated and release cytokines, which reduces glutamate uptake and further impair synapse function. Besides, extracellular glutamate accumulation induced by Aβ mediates synapse toxicity resulting from reduced glutamate receptors and glutamate spillovers. Aβ also mediates synaptic dysfunction by acting on various signaling pathways and molecular targets, disrupting mitochondria and energy metabolism. In addition, Aβ overdeposition aggravates the toxic damage of hyperphosphorylated tau to synapses. Synaptic dysfunction plays a critical role in cognitive impairment of AD. The review addresses the possible mechanisms by which Aβ mediates AD-related synaptic impairment from distant perspectives.

## Introduction

AD is currently considered as a disease of synaptic failure (Babri et al., 2012), which is closely related to β-amyloid (Aβ) deposition and tau hyperphosphorylation (Kopeikina et al., 2012; Breijyeh and Karaman, 2020, Author Anonymous, 2020). Synapses are the locus where information is transferred from a pre-to a postsynaptic neuron (Bellot et al., 2014), which is largely mediated by neurotransmitters that are released by the presynaptic axon terminals and then bind to receptors on the postsynaptic dendritic spines (Raven et al., 2018). The regulation and alteration of synaptic function are generally recognized as synaptic plasticity. Once synaptic plasticity impaired, the synaptic connections and neural communication were disrupted, ultimately causing brain dysfunction at large. For example, the processing and storage of information result from external stimuli were dysfunctional (Zucker and Regehr, 2002; Cooke and Bliss, 2006; Raven et al., 2018). Long-term potentiation (LTP) and long-term depression (LTD) are the two manifestations of synaptic plasticity (Yuste and Bonhoeffer, 2001; De Roo et al., 2008), which are recognized as the biological basis of learning and memory activities at the cellular level (Selkoe, 2002; Yun et al., 2006). LTP and LTD are mainly regulated by N-methyl-D-aspartate receptors (NMDARs) and α-amino-3-hydroxy-5-methyl-4-isoxazolepropionic acid receptor (AMPARs). NMDARs and AMPARs are cationic channels gated by the neurotransmitter glutamate that plays a critical role in excitatory synaptic transmission and synaptic plasticity in the central nervous system (CNS). Under physiological conditions, NMDARs and AMPARs are relevant for LTP and LTD, both of them constitute molecular key mechanisms of hippocampal learning and memory (Cooke and Bliss, 2006). Aβ, a potent neurotoxic peptide, can activate NMDARs to increase excessive inflow of calcium ions (Ca^2+^) and trigger the internalization of NMDARs and AMPARs to suppress LTP and facilitates LTD (O'Riordan et al., 2018; Shankar et al., 2008; Findley et al., 2019), ultimately leading to synapse toxicity and learning-memory deficits seen in AD (Wei J. et al., 2010; Jo et al., 2011; Bergström et al., 2016). One study demonstrated that abnormal synaptic transmission and impaired long-term potentiation (LTP) were often well associated with Aβ plaque formation in the transgenic mouse (Roberson et al., 2011). Similarly, it is reported that Aβ and hyperphosphorylated tau play a synergistic role in NMDAR-mediated synaptotoxicity, the excessive formation of reactive oxygen species (ROS) and oxidative stress (De Felice et al., 2007; Bezprozvanny and Mattson, 2008; Kamat et al., 2016). Recent studies proved that NMDAR activation, excessive Ca^2+^ influxes, and free radical generation are closely related to synaptic dysfunction and tau phosphorylation (Kamat et al., 2013; Rai et al., 2013). As Aβ is an important participant in the pathogenesis of AD, which damages the synaptic plasticity and mediates the synaptic excitotoxicity *via* different mechanisms (Babri et al., 2012). However, the series of events that impair the synaptic function induced by Aβ still under debate. Therefore, in this review, we elaborated the role of Aβ in the synaptic dysfunction related to AD, hoping to find new therapeutic targets for AD through a further insights into the pathological mechanism of AD.

## Aβ can disrupt glutamate receptors to damage synapse plasticity

**FIGURE 1:**
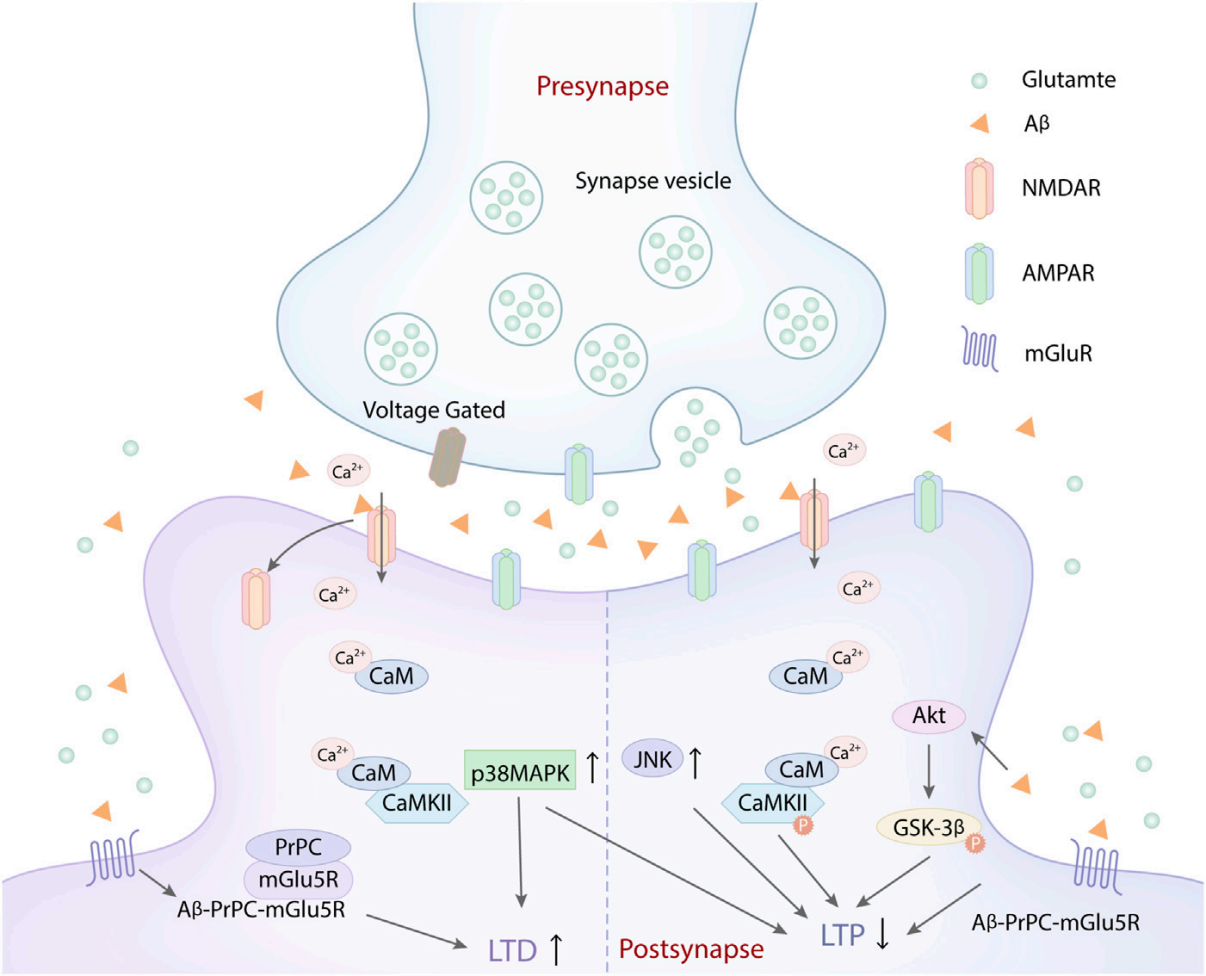
Aβ disrupts synapse plasticity by acting on glutamate receptors. Aβ activated NMDARs and increased Ca^2+^ influx, which leaded to intracellular calcium overload and suppressed the LTP. Besides, the glutamate released from synapse vesicle in presynapse also increased the activation of NMDARs and AMPARs, which aggravated the intracellular Ca^2+^ overload, further inhibiting the LTP. Aβ peptide triggered the rapid activation of Ca^2+^ and CaMKІІ. The aberrant activation of CaMKІІ causes deficits in LTP, leading to synaptic loss *via* the destabilization of AMPARs. Similarly, Aβ could selectively impair glutamatergic synaptic transmission, LTP and LTD expression by activating p38 MAPK and JNK. At the same time, the aberrant activation of Akt induced byAβ can promote the phosphorylation of GSK-3β, which also inhibited LTP. In addition, Aβ assemblies acted on cellular prion protein (PrPC) and mGlu5R, which induced the formation of the complex of Aβ-PrPC-mGlu5R triad at the postsynaptic density. The complex formation caused the mGlu5R-dependent LTD enhancement and NMDAR-dependent LTP inhibition, which exacerbated the damage of synaptic plasticity.

Aβ is one of the main pathological factors of AD (Knobloch et al., 2007; Tu et al., 2014; Herrup, 2015). Aβ can not only damage synaptic function *via* its own internalization and intracellular accumulation but also disrupt synaptic glutamatergic receptors, NMDA and AMPA receptors, which mediate synaptic plasticity impairment in the early stage of AD (Ripoli et al., 2014). Aβ oligomers can activate NMDA and AMPA receptors to disrupt calcium homeostasis, which interference with the main forms of synaptic plasticity, LTP and LTD (Takahashi et al., 2002; Tu et al., 2014) (Figure 1). Studies indicated that Aβ oligomers could directly or indirectly act on NMDA and AMPA receptors, dysregulate their activity, disrupt calcium influx, which resulted in LTP suppression and LTD enhancement (Shankar et al., 2007; Shankar et al., 2008; Tu et al., 2014). Many synaptic membrane receptors are the major sites of Aβ toxicity, especially mGluR and NMDARs (Ittner and Götz, 2011; Yang et al., 2018). The dysregulation of NMDARs induced by Aβ is more prominent in synaptic plasticity damage. Excessive or inappropriate activation of NMDARs mediated by Aβ can inhibit LTP (Coan et al., 1989). However, synaptic and extrasynaptic NMDARs activation have different consequences on synaptic plasticity, gene regulation and neuronal death (Léveillé et al., 2008; Li et al., 2011). Aβ oligomers can not only downregulate the synaptic NMDARs function *via* increasing the endocytosis of NMDARs, but also activate extrasynaptic NMDARs to inhibit LTP (Hu et al., 2017). The activation of synaptic NMDARs reduces Aβ generation, while the activated extrasynaptic NMDARs increases Aβ production. One study showed that soluble Aβ_1-42_, a toxic peptide, which significantly increased the extrasynaptic NMDA response and Ca^2+^ inflow through extrasynaptic NR2B-containing NMDARs (Zhou et al., 2010; Yang et al., 2018), ultimately impaired spatial cognitive function and inhibited LTP (Zhou et al., 2010). Extrasynaptic NR2B-containing NMDA receptor might be a target which mediates the early neuronal dysfunction induced by soluble Aβ_1-42_ (Sinor et al., 2000). Aβ_1-42_ can accelerate the phosphorylation of GluN2B subunits tyrosine and trigger an ifenprodil-sensitive transient activation of Akt (Abbott et al., 2008). Akt Activation mediates the phosphorylation of glycogen synthase kinase-3β (GSK-3β), which obviously interferes with the induction of synaptic plasticity, including LTP (Figure 1) (Hooper et al., 2007; Peineau et al., 2007). However, LTP suppression mediated by soluble Aβ_1-42_ was mainly attributed to the overactivation of extrasynaptic NR2B-containing NMDA receptors, which further disrupted calcium homeostasis and damaged synaptic plasticity (Li et al., 2011). Besides, another study showed that LTP inhibition was more affected by Ca^2+^ influx induced by the activation of GluN2A-rather than GluN2B-containing NMDARs (Izumi et al., 2006; Hu et al., 2009). Aβ may act on multiple subunits of NMDARs, including NR2A and NR2B. Furthermore, NMDARs overactivation and LTP suppression induced by Aβ may be a vicious circle, which exacerbated the damage of synaptic plasticity.

In addition, one study demonstrated that oligomeric and synaptotoxic forms of the Aβ peptide could trigger the rapid activation of Ca^2+^ and calmodulin-dependent protein kinase ІІ (CaMKІІ) (Figure 1). The aberrant activation of CaMKІІ causes deficits in LTP, leading to synaptic loss *via* the destabilization of AMPARs (Takumi et al., 1999). It is well known that the phosphorylation of GluR1-containing AMPARs at a CaMKІІ-dependent site could facilitate LTP generation (Lisman et al., 2002). Aβ-induced synaptic dysfunction is associated with the removal of synaptic AMPARs (Zhao et al., 2004; Gu et al., 2009). Therefore, oligomeric Aβ can impair synapse plasticity by disrupting glutamatergic receptors and calcium homeostasis (Reinders et al., 2016; Arbel-Ornath et al., 2017; Liu et al., 2019). However, the effect of oligomeric Aβ on glutamatergic receptors may be bidirectional. Similarly, the effect of Aβ on synaptic plasticity was concentration-dependent (Falcicchia et al., 2020). At physiological levels, Aβ selectively enhances NMDAR-mediated currents and synaptic transmission (Kelly and Ferreira, 2006; Domingues et al., 2007). Brief periods of high synaptic activity open NMDARs, increasing postsynaptic AMPARs, spine growth, and LTP of synaptic transmission (Hayashi et al., 2000; Kopec et al., 2006). While increased Aβ application promotes endocytosis of NMDARs in cortical neurons and produces a rapid and persistent depression of NMDA-evoked currents in cortical neurons *via* activating nAChRs (Snyder et al., 2005). Under pathological condition, high concentrations of Aβ can enhance the activation of NMDARs (Kawamoto et al., 2008) and cause NMDAR agonist-induced delayed cognitive dysfunction (Nakamura et al., 2006). Low concentration of Aβ can selectively impair glutamatergic synaptic transmission, LTP and LTD expression by activating p38 MAPK and JNK (Origlia et al., 2008; Origlia et al., 2010; Falcicchia et al., 2020) (Figure 1). Thus it can be seen that increased Aβ production increases NMDAR activation, and increased NMDAR activation in turn increases Aβ production within limits. Once the homeostatic balance between NMDA activation and Aβ production is broken, NMDAR overactivation or Aβ deposition both can damage synaptic plasticity, ultimately leading to cognitive impairment in AD (Parihar and Brewer, 2010). Additionally, low-dose Aβ can accelerate the generation of LTD mediated by metabotropic glutamate-5 receptors (mGlu5Rs). mGlu5Rs is a primary glutamate receptor subtype, which also participates in LTP inhibition induced by Aβ (Hu et al., 2014). Besides, certain synaptotoxic Aβ assemblies act on cellular prion protein (PrPC), which is involved in LTD generation mediated by Aβ (Laurén et al., 2009; Nicoll et al., 2013). Aβ, PrPC and mGlu5R can form the complex of Aβ-PrPC-mGlu5R triad at the postsynaptic density. Aβ-PrPC-mGlu5R triad not only enhances mGlu5R-dependent LTD, but also inhibits NMDAR-dependent LTP (Figure 1), which exacerbates the damage of synaptic plasticity (Um et al., 2013). Therefore, targeting Aβ-PrPC-mGlu5R triad may provide a new direction for preventing synaptic plasticity damage in early AD.

## Aβ can destroy glutamate cycle to induce synapse toxicity

**FIGURE 2:**
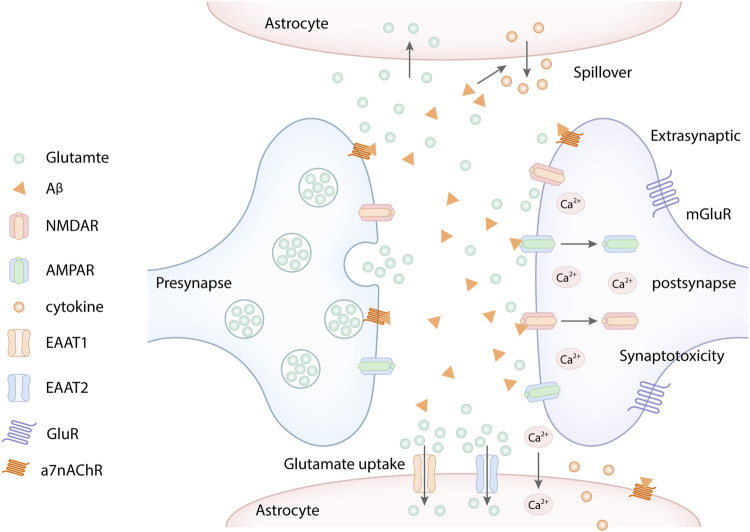
Aβ promotes the extracellular glutamate aggregation. Aβ increased the glutamate spillover through α7 nicotinic acetylcholine receptors (a7nAChR), which excessively activates extrasynaptic NMDARs, subsequently resulting in Ca^2+^ influx. Similarly, Aβ overexpression mediated the endocytosis of NMDA and AMPA receptors, which further increased the extracellular glutamate aggregation. In addition, Aβ reduced neuronal glutamate uptake by decreasing glutamate transporter EAAT1 and EAAT2 in astrocytes, which decreases glutamate clearance. Besides, Aβ oligomers can activate astrocytes, which released pro-inflammatory cytokines and inhibited the ability of glutamate uptake of astrocytes.

Aβ oligomers can induce extracellular glutamate accumulation, which destroys the glutamate cycle and results in the synapse toxicity (Li and Selkoe, 2020). The extracellular glutamate accumulation mainly blames on Aβ-induced dysregulation of two mechanisms. On the one hand, Aβ can reduce neuronal glutamate uptake by triggering the rapid glutamate transporter mislocalization and internalization in astrocytes (Matos et al., 2008; Li et al., 2009; Matos et al., 2012; Lanznaster et al., 2017), which decreases glutamate clearance (Scimemi et al., 2013; Yang et al., 2018). On the other hand, Aβ can increase the glutamate spillover through α7 nicotinic acetylcholine receptors (a7nAChR) (Kabogo et al., 2010; Hascup and Hascup, 2016), which excessively activates extrasynaptic NMDARs, subsequently resulting in neurotoxicity (Varga et al., 2014). Studies have demonstrated that Aβ oligomers could facilitate the release of glutamate by activating astrocytes through a7nAChR, thereby enhancing the activation of extracsynaptic NMDARs (Talantova et al., 2013) (Figure 2). Similarly, the aggregation of extracellular glutamate induced by Aβ is recognized as a detrimental upstream factor, which can also cause the overactivation of extrasynaptic NMDARs, ultimately leading to LTP impairment, LTD enhancement, and synapse loss (Huang et al., 2018). In addition, Aβ oligomers can combine with the fibronectin repeats domain of EphB2 and trigger EphB2 degradation in the proteasome. EphB2 can regulate the synaptic localization of NMDARs, which mediates tyrosine phosphorylation of GluN2B at Y1472 and stabilizes NMDARs on the cell surface and thereby enhances the response of NMDARs. GluN2B phosphorylation at Y1472 is important for GluN1/GluN2B trafficking to the cell member, which plays an important role in NMDA receptor-dependent synaptic plasticity (Salter and Kalia, 2004; Shi et al., 2016). Aβ oligomers can inhibit GluN2B phosphorylation and the surface expression of GluN2B *via* meidating EphB2 degradation in the proteasome (Hu et al., 2017). At the same time, Aβ oligomers can accelerate NMDARs endocytosis result from reducing GluN2B phosphorylation. It can also activate extrasynaptic NMDARs to inhibit LTP (Hu et al., 2017). Besides, Aβ can decrease the surface expression of GluN1 in cortical neurons by activating a7nAChR and the tyrosine phosphatase STEP (Snyder et al., 2005).

Some studies have proved that oligomeric and synaptotoxic forms of the Aβ peptide induced aberrant activation of CaMKII, which leaded to deficits in LTP, ultimately resulting in synaptic loss *via* the destabilization of AMPARs (Lisman et al., 2002). It is well known that excitatory synapses contain AMPA and NMDA ionotropic glutamate receptors as well as metabotropic type glutamate receptors (mGluRs) positioned on dendritic spines (Snyder et al., 2005; Hsieh et al., 2006). Aβ-induced synaptic dysfunction plays a critical role in the synaptic removal of AMPARs (Gu et al., 2009). In addition, Aβ overexpression can mediate the endocytosis of NMDA and AMPA receptors (Figure 2), which inhibits NMDA and AMPA receptor-mediated synaptic transmission, leading to synapse toxicity (Snyder et al., 2005; Hsieh et al., 2006; Yang et al., 2018). One study showed that Aβ_1-42_ preferentially binds to glutamatergic neurons expressing NR1 or NR2B-containing NMDA receptors compared with other subunits (Lacor et al., 2007). The decreased NMDARs and AMPARs induced the glutamate aggregation, which in turn increased the endocytosis and decreased surface expression of NR1 and NR2B (Snyder et al., 2005; Findley et al., 2019). Similarly, Aβ can activate the apoptotic effector component caspase-3, which is required in AMPAR removal and consequent LTD induction (Li et al., 2010). Reduced glutamate receptors further increase extra-synaptic glutamatergic accumulation, subsequently result in Aβ-induced synaptotoxic effects (Snyder et al., 2005). Aβ disrupts the glutamatergic transmission system by significantly decreasing the levels of AMPARs and NMDARs at the neuronal plasma membrane (Roselli et al., 2005; Lacor et al., 2007; De Felice et al., 2009). In a word, Aβ oligomers can aggravate the glutamate excitotoxicity by damaging glutamate transporters resulting from interrupting glutamate receptors, including NMADRs, AMPARs, and metabotropic glutamate receptors (Li and Selkoe, 2020).

Moreover, Aβ oligomers can aslo inhibit the astrocytic glutamate uptake by decreasing astrocytic glutamate transporters expression, causing extracellular glutamate aggregation, ultimately leading to a serious of glutamate toxicity cascades (Fernández-Tomé et al., 2004; Matos et al., 2008; Tong et al., 2017; Huang et al., 2018). Besides, Aβ oligomers can activate glial cells, which can release pro-inflammatory cytokines in pathological conditions. The pro-inflammatory cytokines can inhibit the ability of glial glutamate uptake and impair glutamate transporters (Carmen et al., 2009; Dumont et al., 2014; Tong et al., 2017). Consistent with these results, both glutamate transporters EAAT1 and EAAT2 which play an important role in the glutamate uptake in glial cells are decreased in the hippocampus of AD patients (Jacob et al., 2007). In a word, Aβ can disrupt Glu-recycling at the synapse by increasing glutamate spillover or decreasing glutamate transporters (Varga et al., 2015) (Figure 2). Taken together, Aβ can disrupt Glu-recycling at the synapse by increasing glutamate spillover, decreasing glutamate uptake, and impairing glutamate transporters, causing the accumulation of excessive glutamate in the extrasynaptic space, which in turn aberrantly activates eNMDARs and induces synaptic dysfunction.

## Aβ can disturb molecular signaling pathway to damage synapse

Aβ oligomers not only act directly or indirectly on glutamate receptors, but also interfere with synapse-related signaling pathways and molecular targets to impair synaptic function. Studies showed that Aβ-induced dsyregulation of NMDARs inhibits the Wnt/β-catenin signaling pathway (Inestrosa et al., 2007; Magdesian et al., 2008). Under normal physiologic condition, the activation of Wnt signaling pathway can facilitate GSK-3β inactivation, elevate intracellular β-catenin, and promote Wnt target gene transcription, which are conducive to dendrite development, synapse formation, glutamate receptor insertion, and synaptic plasticity in the postsynaptic region (Beaumont et al., 2007; Cerpa et al., 2011; Inestrosa and Varela-Nallar, 2014). Some studies indicated that the activation of Wnt-5a signaling pathway protected PSD-95 from Aβ-induced synaptic toxicity and activated downstream proteins such as PKC, CaMKII, and JNK (Kühl et al., 2000; Dinamarca et al., 2008; Killick et al., 2014). Among of them, the Wnt-5a/JNK pathway modulates PSD-95 (Farías et al., 2009; Varela-Nallar, et al., 2010); the Wnt-5a/PKC pathway directly adjusts the localization of NR1 on the postsynaptic membrane (Frozza et al., 2009; Cerpa et al., 2011). At the same time, alteration of CaMKII activity triggers the incorporation of NMDARs in the synapse (Kühl et al., 2000). Therefore, the loss of Wnt signaling is closely related to the neurodegeneration and synaptic impairment induced by Aβ in AD (Liu et al., 2014). Aβ-induced neurotoxicity causes GSK-3β activation and decreases β-catenin (Chen et al., 2000; Magdesian et al., 2008), which cause reduced expression of Wnt-target gene and inhibition of Wnt signaling pathway (Cerpa et al., 2010; Cerpa et al., 2011). One study proved that the GSK-3β expression was up-regulated and the β-catenin was down-regulated in the hippocampus of AD patients (Chen et al., 2000). Besides, Aβ-induced GSK-3β overactivation increases the endocytosis/internalization of NMDARs and AMPARs, which disrupts the glutamatergic transmission and damage the synaptic function (Chen et al., 2007; Wei W. et al., 2010; Deng et al., 2014). Moreover, Aβ oligomers also attributes to the reduction and degradation of post-synaptic density-95 (PSD-95), which is a vital protein to sustain synaptic plasticity and the stabilization of AMPAR and NMDAR at synapses (Roselli et al., 2005). Once NMDAR and AMPAR becomes dysfunctional, it can lead to neuronal Ca^2+^ overload, oxidative stress (De Felice et al., 2007), and inhibition of the Wnt/β-catenin signaling pathway (Inestrosa et al., 2007; Parihar and Brewer, 2010) (Table 1).

**TABLE 1:** Aβ disrupts synapse-related signaling pathways and molecular targets.

Signaling pathways/molecular targets	Inhibit/activate	Result	References
Wnt/β-catenin	Inhibit	Cause neurodegeneration and synaptic impairment	Liu et al. (2014); Parihar and Brewer, (2010); Inestrosa et al. (2007)
IKK/NF-κB	Activate	Mediate neuroinflammation and memory impairment	Heppner et al. (2015); Wang et al. (2005)
JAK2/STAT3	Inhibit	Aggravate memory disorder	Chiba et al. (2009)
JNK	Activate	Inhibit NMDA and AMPA responses	Chergui et al. (2004); Peng et al. (2013)
Akt	Activate	Inhibit LTP	Jo et al. (2011)
MAPK	Inhibit	Cause AMPARs internalization and synapse collapse	Wang Q. et al. (2004a)
caspase-3	Activate	Inhibit LTP	Jo et al. (2011)
GSK-3β	Activate	Increases the endocytosis/internalization of NMDARs and AMPARs	Chen et al. (2007); Wei J. et al. (2010a); Deng et al., 2014
CDK-5	Activate	Inhibit NMDA and AMPA responses	Chergui et al. (2004); Peng et al. (2013)

Abbreviations:Aβ, amyloid-beta; CDK-5, cyclin-dependent kinase 5; AMPA, α-amino-3-hydroxy-5-methyl-4-isoxazolepropionic acid; CGC, cerebellar granule cell; GSK-3β, glycogen synthase kinase-3β; JAK2, Janus kinase 2; JNK, c-Jun N-terminal kinase; LTP, long-term potentiation; MAPK, mitogen-activated protein kinase; NMDA, N-methyl-D-aspartate; NMDAR, N-methyl-D-aspartate receptor; STAT3, Signal transducer and activator of transcription 3.

In addition, the IKK/NF-κB signaling pathway also plays a critical role in Aβ-mediated hippocampal LTP impairment. Aβ might alter IKK/NF-κB activity *via* interacting with tumor necrosis factor receptor (TNFR). Aβ can motivate pro-inflammatory cytokine like TNF-α or interleukins to impair memory *via* directly combining with TNF-α receptors or activating microglial cell (Wang et al., 2005; Heppner et al., 2015) (Table 1). Microglia activated by Aβ and TNF-α can induce neuronal cell death (Floden et al., 2005). Besides, TNF-α activates dsRNA-dependent protein kinase (PKR), which mediate neuroinflammation and memory impairment induced by Aβ oligomers. Similarly, overmuch TNF-α also cause the abnormal trafficking of AMPARs, which leads to glutamate excitotoxicity (Leonoudakis et al., 2004). TNF-α acts through TNFR and mediates cellular functions primarily through NF-κB and IKK (Sakurai et al., 2003; Kwon et al., 2004; Luo et al., 2005; Rossol et al., 2007). The activation of NF-κB accelerates pro-inflammatory genes transcription and triggers the expression of Amyloid precursor protein (APP) gene, which results in Aβ overdeposition and synaptic loss (Valerio et al., 2006). Therefore, oligomeric Aβ might suppress hippocampal LTP *via* acting on TNF-α receptor and subsequently activating IKK and NF-κB signaling, ultimately forming a vicious cycle of synaptic damage (Samidurai et al., 2018). In addition, the inhibitory effects of oligomeric Aβ on hippocampal LTP involves apoptotic proteins (caspases) and reactive oxygen species (ROS) production (Ivins et al., 1999; Wang et al., 2004a). Oligomeric Aβ can intervenes in hippocampal LTP by activating caspase-3, AKT8 virus oncogene cellular homolog (Akt), and GSK-3β pathway (Jo et al., 2011).

Besides, soluble Aβ can aggravate memory disorder resulting from interfering with the Janus kinase 2 (JAK2)/Signal transducer and activator of transcription 3 (STAT3) axis and cholinergic dysfunction (Chiba et al., 2009). Kinases JNK, CDK-5, p25, and p38 MAPK are also involved in LTP inhibition mediated by Aβ (Wang et al., 2004b; Danysz and Parsons, 2012). p25 is a molecule that can induce the hallmark early Alzheimer-like synaptic pathology. Recent studies showed that stimulation of CDK-5 and JNK signaling by overproduction of p25 can rapidly inhibit NMDA and AMPA responses, reduce synapse density *via* the removal of newly delivered synaptic AMPARs, which altered synaptic transmission (Chergui et al., 2004; Peng et al., 2013). The up-regulation of JNK/Aβ induced synaptic depressions, which has the same result as suppressing and eliminating synapses (Zhu et al., 2005; Sheng et al., 2016) (Table 1). Besides, Aβ can also mediate synaptic depression on account of mGluRs activation, which provoked a series of downstream molecular events including MAPK and calcineurin, ultimately caused AMPARs internalization and synapse collapse (Wang, Rowan et al. (2004a)). In brief, Aβ can interfere with distant molecular signaling pathways to destroy synaptic transmission and plasticity.

## Aβ can damage mitochondria and energy metabolism

Mitochondria provide the necessary ATP for the survival and optimal function of neurons, and mitochondrial dysfunction is closely related to aging and neurodegenerative diseases (Scheibye-Knudsen, et al., 2015; Kerr, et al., 2017). In AD, mitochondrial dysfunction has been widely recognized as a common pathological hallmark (Silva et al., 2011; Sorrentino, et al., 2017). The overexpression of APP and Aβ deposition can cause mitochondrial malfunctioning, such as mitochondrial fragmentation and abnormal distribution, which results in abnormal mitochondrial dynamics and ATP synthesis (von Bernhardi and Eugenín, 2012). Excessive mitochondrial fission and increased ROS production results in the progression of extensive macromolecular oxidative damage and amyloid lesions caused by ROS (Yu et al., 2006; Lakatos et al., 2010). Moreover, the interaction between intracellular Aβ and 3-hydroxyacyl-CoA dehydrogenase type-2 (HSD17B10, also known as ABAD) further lead to mitochondrial dysfunction and ROS leakage (Lustbader et al., 2004). Meanwhile, Aβ can interact with dynamin-1-like protein Drp1 (DNM1L), which can also promote excessive mitochondrial fragmentation and neuronal damage (Manczak et al., 2011). The mitochondria impairment alters the mitochondrial membrane potential, dysregulates mitochondrial calcium levels, and increases mitochondrial O^2-^ (Chung et al., 2001). In addition, mitochondrial dysfunction-induced energy deficiency and Aβ_1-42_ oligomers cause intracellular Ca^2+^ imbalance and 5′AMP-activated protein kinase (AMPK) activation, resulting in synaptotoxicity and memory loss (Mairet-Coello, et al., 2013; Kerr, et al., 2017). The dysregulation of Ca^2+^ homeostasis further causes mitochondrial dysfunction, impairments in synaptic transmission and plasticity, and oxidative stress, which leads to the age-related cognitive impairment (Kang et al., 2011). Moreover, the axonal transport of mitochondria play a key role in neuronal function, Aβ_1-42_ and phosphorylated tau mediate defects in axonal transport, leading to synapse starvation, ATP depletion, and ultimately neurodegeneration (Vossel, et al., 2010; Fang, et al., 2019). Therefore, there may be a series of complex relationships between mitochondrial dysfunction and AD, suggesting that targeting defective mitochondria may be an important approach for AD treatment.

## Aβ and tau have synergistic toxic effects on synapses

**FIGURE 3:**
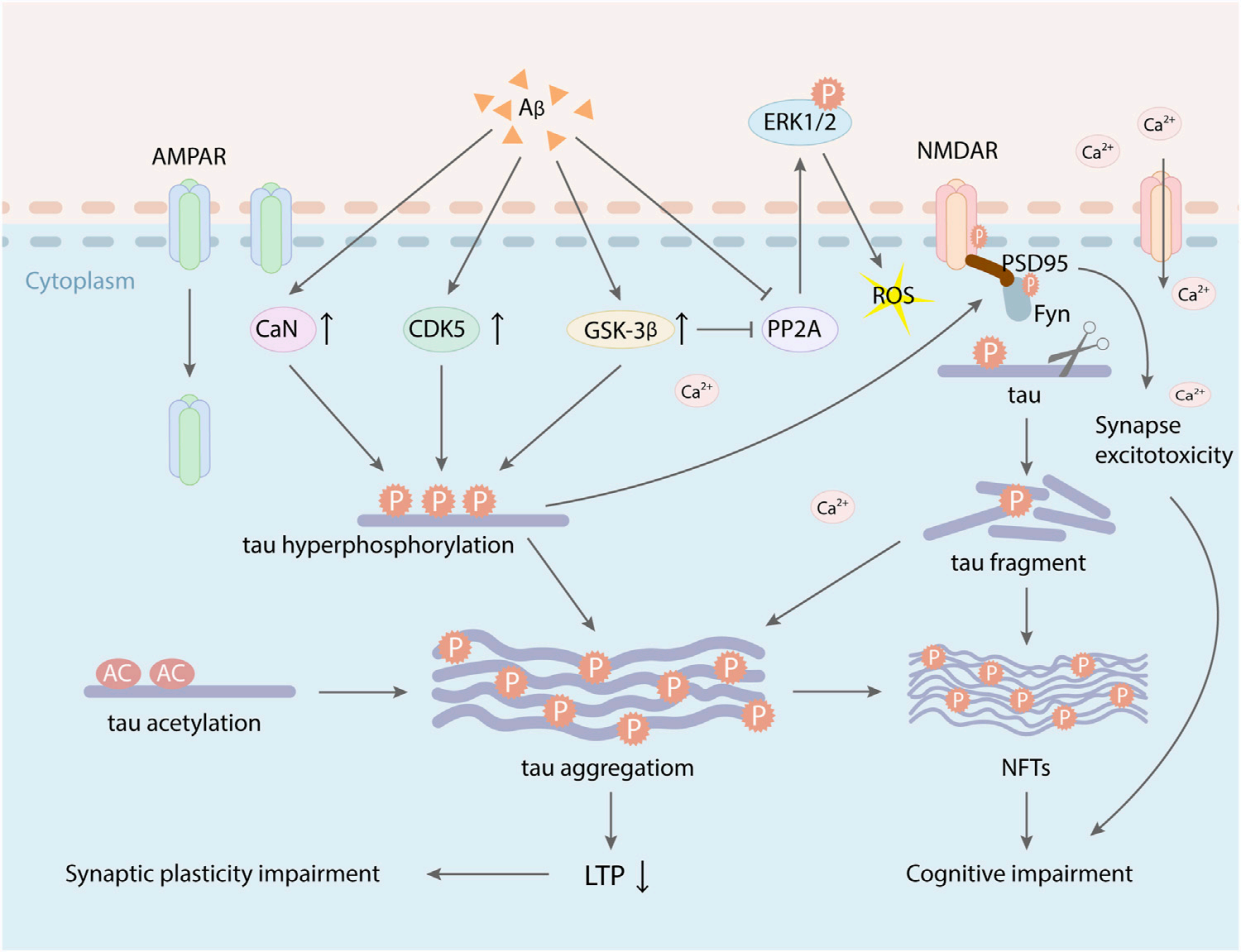
Aβ and tau have synergistic toxic effects on synapse plasticity. Excessive deposition of Aβ increased the activity of GSK-3β, CDK-5, and CaN, which promoted tau hyperphosphorylation and the formation of NFTs, leading to LTP suppression and cognitive impairment. Aberrant phosphorylation of tau protein can mediate Fyn facilitation to dendrites and make Fyn to interact with the PSD-95/NMDAR complex, which induced the synapse toxicity. At the same time, activated GSK-3β and tau hyperphosphorylation also induced AMPAR internalization. PP2A inactivity involved in hyperphosphorylation of tau and facilitated phosphorylation of extracellular receptor kinase (ERK) 1/2, which caused ROS and oxidative stress. Besides, Aβ induced tau acetylation, which increased tau aggregation and also inhibited the expression of LTP.

Aβ and tau are two main pathogenic factors of AD. Aβ and hyperphosphorylated tau have synergistic effect on impairing synapse function. Under physiologic condition, tau proteins increase in the PSD-enriched fraction accompanied by enrichment in PSD-95 and GluA1, which are requisite for synaptic potentiation related to LTP (Ehlers, 2003; Steiner et al., 2008; Kessels and Malinow, 2009). However, hyperphosphorylated tau disrupts NMDARs and AMPARs trafficking and anchoring (Hoover et al., 2010; Ittner et al., 2010; Miller et al., 2014), which can reduce synaptic responses and inhibit the expression of AMPARs and NMDARs in dendritic spines (Hoover et al., 2010; Crimins et al., 2012; Liao et al., 2014). Recent studies found that reduced NMDA receptor suppressed field excitatory postsynaptic potential (fEPSP) in hippocampal slices of tau transgenic mice (Burnouf et al., 2013). At the same time, tau hyperphosphorylation can decrease the internalization of AMPARs mediated by calcineurin, which reduces postsynaptic transmission at a very early stage of pathology (Miller et al., 2014; Müller-Thomsen et al., 2020). Excessive deposition of Aβ can promote tau hyperphosphorylation and activate GSK-3β, which exacerbates the toxic effect of phosphorylated tau on synapse, ultimately resulting in forming neurofibrillary tangles (NFTs) and loss of neurons and synapses (Wang and Mandelkow, 2016). Hyperphosphorylated tau proteins mediated by Aβ are more prone to hydrolysis or misfolding into tau oligomers. Extracellular tau oligomers acts on the postsynaptic regions, which increases Ca^2+^ influx and damages LTP (Usenovic et al., 2015; Fá et al., 2016). Moreover, tau oligomers also seed the misfolding and aggregation of cellular monomeric tau in a prion-like manner, which cause the diffusion of tau toxicity among synapses, eventually leading to synaptic dysfunction and the progression of AD lesions (Sanders et al., 2014; Comerota et al., 2019). In addition, a synaptic weaken stimulus mediates Ca^2+^ influx mediates through NMDAR and then activates the serine/threonine kinase GSK-3β. Activated GSK-3β triggers the short-lived phosphorylation of tau (Ser396), which paly a vital role in subsequent AMPAR internalization (Regan et al., 2017). Moreover, tau transports the tyrosine kinase Fyn to dendritic spines and interacts with postsynaptic targeting of Fyn kinase as well as its substrates PSD-95 and NR2B subunit (Ittner et al., 2010; Frandemiche et al., 2014) (Figure 3), which mediates downstream excitotoxic effects of phosphorylated tau (Ittner et al., 2010; Tracy et al., 2016; Regan et al., 2017). One study indicated that reduced tau could ameliorate Aβ-induced toxicity and excitotoxicity *in vivo* (Roberson et al., 2007). The result manifests that the synaptic toxicity of Aβ was dependent on tau to some extent (Biundo et al., 2018). Besides, tau phosphorylation is also regulated by the activities of protein phosphatase-2A (PP-2A), which is decreased in AD (Gong et al., 1993; Gong et al., 2000). Aβ can inhibit the activation of kinase PP2A (Chan and Sucher, 2001; Chohan and Iqbal, 2006). Similarly, the overexpression of GSK-3β induced by Aβ also inhibits PP2A, which may appear a negative feedback mechanism for GSK-3β activity (Liu et al., 2008). PP2A inactivity is involved in hyperphosphorylation of tau and facilitates phosphorylation of extracellular receptor kinase (ERK) 1/2 (Schulz et al., 2014). Therefore, It can be assumed that decreased PP2A activity may induce the activation of ERK1/2 and several other kinases and the abnormal hyperphosphorylation of tau. Additionally, ERKs can be activated by mitogens, c-Jun N-terminal kinase (JNK)/SAPK, and p38 mitogen-activated protein kinase (MAPK), which belong to stress-activated kinases (Tibbles and Woodgett, 1999). The activities of these enzymes can be triggered by a mass of exogenous and endogenous stress-inducing stimuli, such as ROS and oxidative stress. The kinases that phosphorylate tau can be activated by NMDA-induced oxidative stress including hyperactivation of CDK5 signaling pathway, MAPK, and several stress-activated protein kinases (Fuentes et al., 2012). Tau phosphorylation mediated by these kinases impaired microtubule assembly and induced the formation of paired helical filament (PHF) (Evans et al., 2000). Aβ activates GSK-3β, which induces oxidative stress, hyperphosphorylation of tau, NFT formation, neuronal death, and synaptic loss that can induce memory deficits. Furthermore, Aβ can activate calcineurin (CaN) to inhibit NMDAR function and impair LTP (Figure 3). Activated CaN increases tau phosphorylation, neurodegeneration, tangle formation and, subsequently, synapse dysfunction ((Ermak et al., 2001; Cook et al., 2005).

Studies demonstrated that the accumulation and mislocalization of hyperphosphorylated tau in the somatodendritic compartment of neurons in AD interfered with glutamate receptor trafficking and synaptic function (Ballatore et al., 2007; Hoover et al., 2010). Tau protein also combines with a postsynaptic protein complex including PSD-95, the scaffold for synaptic NMDARs which can adjust synaptic plasticity (El-Husseini et al., 2000). Endogenous tau protein is typically present in the post-synaptic dendrites, where it interacts with the PSD-95/NMDAR complex (Mondragón-Rodríguez et al., 2012). Treatment with human tau into the presynaptic terminal disrupts synaptic transmission, which may be closely related to blocking proper docking of synaptic vesicles (Moreno et al., 2011). Some studies also reported that tau toxicity on synaptic plasticity was involved in the acetylation of two lysines on tau, K274, and K281, which were related to AD. Acetylation decreased tau degradation (Min et al., 2010), inhibited tau binding to microtubule (Cohen, et al., 2011; Sohn et al., 2016), and increased tau aggregation (Cohen, et al., 2011; Min et al., 2015). Acetylated tau could inhibit the expression of LTP and lead to dysmnesia in AD (Figure 3). At the same time, acetylated tau could also disrupt the postsynaptic localization of memory-related KIBRA, which restrained the recruitment of postsynaptic AMPARs and damaged synaptic plasticity (Zempel et al., 2010; de Calignon et al., 2012; Tracy et al., 2016; Tracy and Gan, 2017). Therefore, tau may play an important role in regulating synaptic function and targeting neurotransmitter receptors to the synapse (Ittner and Götz, 2011; Kamat et al., 2016), while Aβ may be a synergist in this event.

## Conclusion and future directions

Synaptic impairment is an early lesion of AD. Aβ, as one of major pathogenic factors in AD, is involved in the early synaptic damage. On the one hand, Aβ can disrupts extrapsynaptic/synaptic NMDARs to mediate Ca^2+^ influx, which leads to intracellular calcium overload and synaptic damage. Excessive Aβ deposition promotes NMDARs activation, which in turn increases Aβ production to a certain extent. Both can damage synaptic plasticity, which results in cognitive deficit of AD (Parihar and Brewer, 2010). On the other hand, Aβ can induce the internalization or removel of extrapsynaptic NMDARs and AMPARs by distant mechanisms, which results in extracellular accumulation of glutamate. The damage of synaptic plasticity is mainly blamed on calcium dyshomeostasis caused by NMDAR overactivation. The neuroexcitatory toxicity is mainly blamed on glutamate aggregation induced by reduced glutamate receptors and the dysfunction of glial cells induced by Aβ. NMDARs can not only improve learning and memory *via* sustaining LTP, but also cause synaptic plasticity damage and excitotoxicity through excitatory amino acid toxicity induced by NMDARs, resulting in learning and memory impairment, which is associated with Ca^2+^ disorders mediated by NR2B subunit (Martel et al., 2009). Besides, excessive NMDAR activation leads to excessive Ca^2+^ influx, which triggers a series of toxic reaction and activates diverse degradation enzymes, such as phospholipase C, CAMKII, PKC, NO synthase. As a result, they destroy neuronal lipid membrane and opalase bone frame, which disrupts the synaptic transmission, eventually developing learning and memory deficits (Biondi et al., 2010). In a word, Aβ-induced excessive Ca^2+^ influx, extracellular glutamate excitotoxicity, and increased internalization of NMDARs can suppress LTP, ultimately results in neuronal damage and learning-memory impairment in AD (Shankar et al., 2007; Talantova et al., 2013; Findley et al., 2019). In addition, the overexpression of APP and Aβ deposition can cause mitochondrial malfunctioning and destroy energy metabolism. Therefore, reducing Aβ deposition and extrasynaptic NMDARs overactivation may be a worthy consideration for preventing synaptic impairment in AD (Rammes et al., 2011). Meanwhile, targeting defective mitochondria may be an important approach for AD treatment. The current drug memantine which inhibits extrasynaptic NMDAR activity can be considered. Memantine is a non-competitive NMDAR antagonist, which can ameliorated Aβ-induced dysfunction of GluN2B-containing NMDARs trafficking by inhibiting the phosphorylation and surface expression of GluN2B, and prevent the downregulation of ERK/CREB signaling to alleviate neurotoxicity. Excitotoxicity refers to the sustained stimulation of excitatory amino acid receptors mainly involving NMDARs, which mediated a series of toxic impairment to neurons (Shin and Linden, 2005; Nicholls et al., 2007). The series of toxic impairment derived from excitotoxicity is mainly due to the upregulation of detrimental signaling pathways, disrupted Ca^2+^ homeostasis, and ROS/reactive nitrogen species (RNS) with further oxidative/nitrosative stress ultimately leading to cell death (Rai et al., 2013). Collective information suggests that NMDAR-mediated oxidative stress and neuronal apoptosis directly or indirectly influences synapse function (Kamat et al., 2016). The production of Aβ also requires activation of extrasynaptic NMDARs (probably GluN2B-enriched NMDARs) (Bordji et al., 2010). In conclusion, the glutamate-induced excitotoxicity and synaptic dysfunction could be excellent targets for the therapy of AD (Kamat et al., 2016). In addition, Aβ promotes tau phosphorylation in different manners, which further mediates the synaptic toxicity. Thus, blocking the targets of synergistic effect of Aβ and tau needs to be further explored, which may provide a new insight to prevent AD.
